# Supplementary material for: Compliance to Multidisciplinary Lifestyle Intervention Decreases Blood Pressure in Patients with Resistant Hypertension: A Cross-Sectional Pilot Study
Source: J Clin Med. 2023 Jan 15;12(2):679. doi: 10.3390/jcm12020679 (PMC9867179; doi:10.3390/jcm12020679)
Supplement: Supplementary file 1 [file jcm-12-00679-s001.zip › jcm-2082889-supplementary/Supplementary/Supplementary Table S1.pdf]

Supplementary Table S1. Laboratory assessments in blood and urine.

	Baseline	After intervention	p-value
**Blood**			
Renin (ng/(mL h))	3.3 ± 0.7	5.9 ± 1.5	**0.005**
Aldosterone (nmol/L)	0.6 ± 0.1	0.6 ± 0.05	0.874
ACTH (pmol/L)	4.7 ± 0.5	4.1 ± 0.3	0.157
Creatinine (µmol/L)	91.8 ± 6	94.2 ± 4.8	0.431
Estimated GFR (mL/min/1.73 m^2)	75,9 ± 17,4	73,2 ± 14,5	0.213
Uric acid (mmol/L)	0.4 ± 0.02	0.4 ± 0.02	0.092
Sodium (mmol/L)	139.8 ± 0.4	139 ± 0.5	0.096
Potassium (mmol/L)	4 ± 0.1	4.1 ± 0.1	0.375
Calcium (mmol/L)	9.8 ± 0.4	9.5 ± 0.2	0.172
Phosphorus (inorganic) (mmol/L)	1.1 ± 0.04	1.1 ± 0.03	0.375
Hemoglobin (g/L)	148.2 ± 4	146 ± 0.3	0.519
ESR (mm/H)	26.5 ± 4	27.4 ± 3.5	0.784
CRP (mg/dL)	0.5 ± 0.1	0.4 ± 0.1	**0.032**
Glucose (mmol/L)	6.9 ± 0.5	6.3 ± 0.5	0.211
HbA1c (% of total Hb)	6.5 ± 0.3	6.4 ± 0.3	0.428
PTH (ng/L)	79.5 ± 11.2	92.2 ± 15.9	0.221
Vitamin D3 (nmol/L)	47.2 ± 4.3	54.2 ± 4.6	0.153
LDL-cholesterol (mmol/L)	3 ± 0.1	3 ± 0.2	0.885
HDL-cholesterol (mmol/L)	1.1 ± 0.03	1.2 ± 0.04	0.234
Triglycerides (mmol/L)	1.9 ± 0.2	1.8 ± 0.3	0.772
Total cholesterol (mmol/L)	4.9 ± 0.1	4.8 ± 0.2	0.483
**Urine**			
Albumin (mg/24h urine)	115.9 ± 61.7	71.6 ± 18.5	**0.008**
Protein (mg/24h urine)	343.7 ± 102.4	149 ± 29	**0.010**
Sodium (mmol/L)	138.3 ± 13.7	167.2 ± 14.2	0.203
Potassium (mmol/L)	69.5 ± 5.2	71.6 ± 5.6	0.628
Metanephrines (µmol/L)	387.7 ± 76.87	519.5 ± 90.5	0.449
UACR (mg/g)	40 (17-100)	23 (7-80)	**0.008**

ACTH: adrenocorticotropic hormone; CRP: C-reactive protein; ESR: erythrocyte sedimentation rate; GFR: glomerular filtration rate; Hb: hemoglobin; HbA1c: glycated hemoglobin; HDL: high density lipoprotein; LDL: low density lipoprotein; PTH: parathyroid hormone; UACR: urine albumin-to-creatinine ratio represented by median and interquartile range.
