# Supplementary figures and images for: Compliance to Multidisciplinary Lifestyle Intervention Decreases Blood Pressure in Patients with Resistant Hypertension: A Cross-Sectional Pilot Study
Source: J Clin Med. 2023 Jan 15;12(2):679. doi: 10.3390/jcm12020679 (PMC9867179; doi:10.3390/jcm12020679)

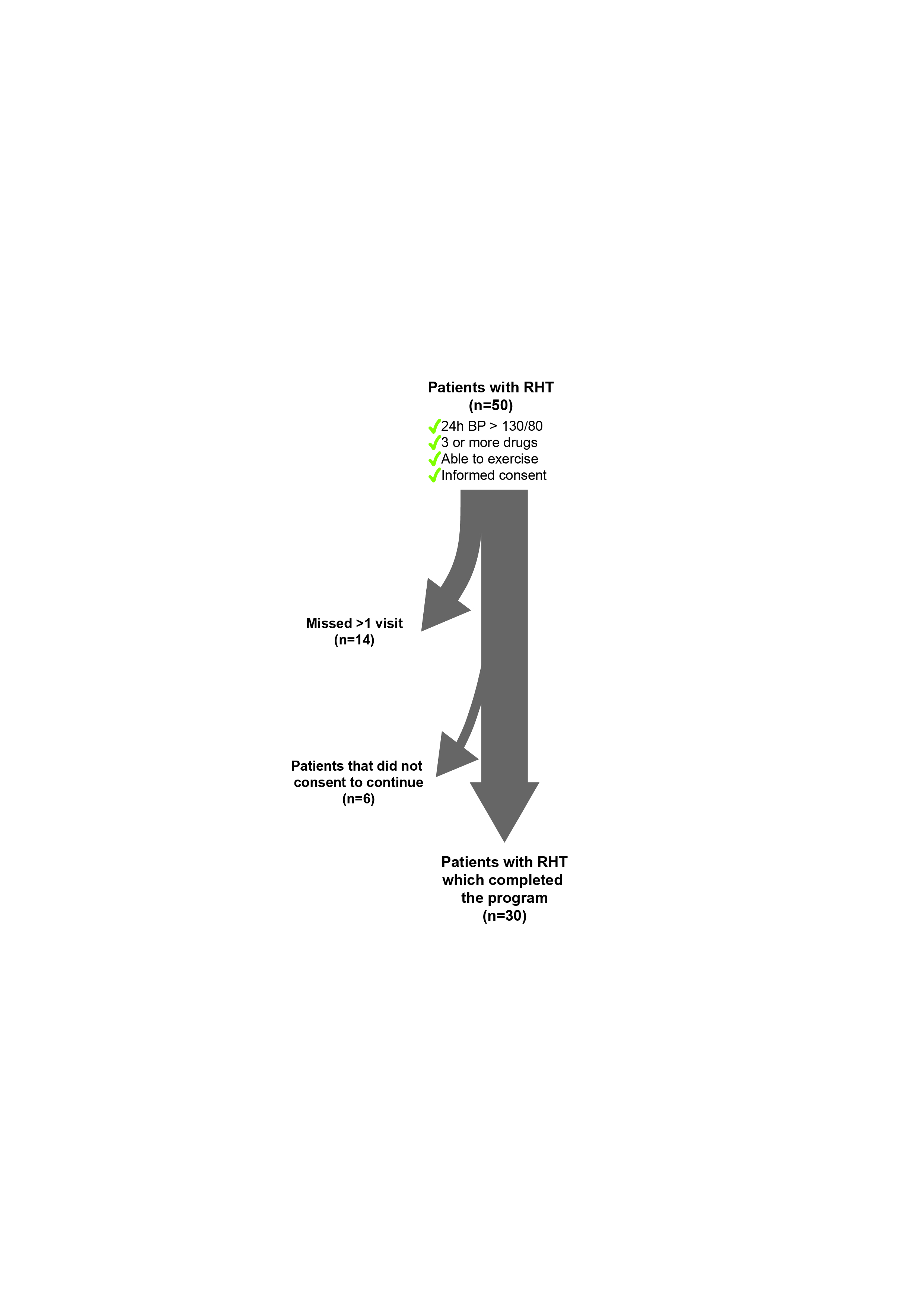

Supplement: Supplementary file 1 [file jcm-12-00679-s001.zip › jcm-2082889-supplementary/Supplementary/Supplementary Figure S1.jpg]

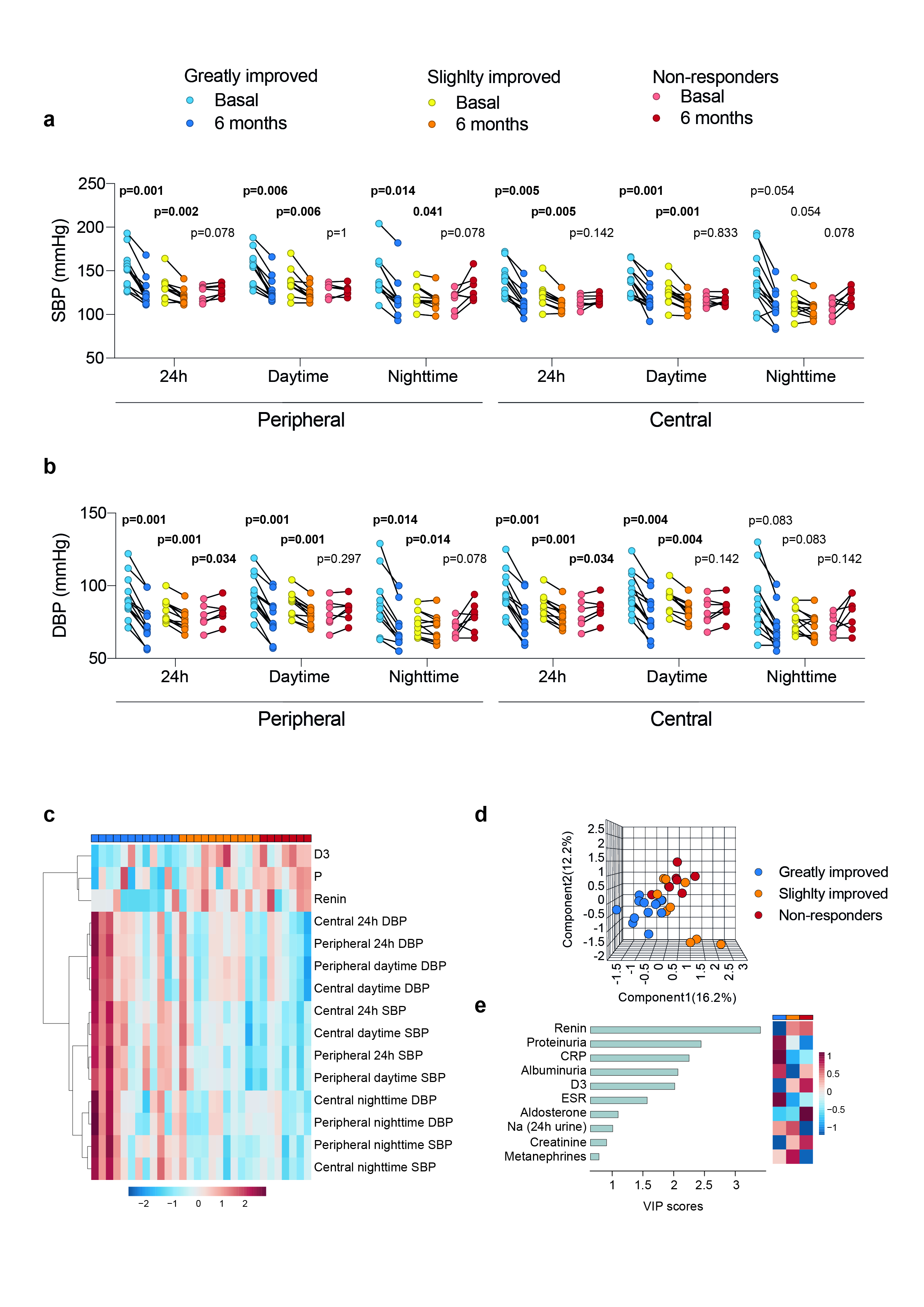

Supplement: Supplementary file 1 [file jcm-12-00679-s001.zip › jcm-2082889-supplementary/Supplementary/Supplementary Figure S2.jpg]
